# A Case of Extreme Bradycardia

**Published:** 1899-06

**Authors:** R. George Fendick, George Parker, F. Richardson Cross


### A CASE OF EXTREME BRADYCARDIA.

BY

R. George Fendick, M.R.C.S., and George Parker, M.D. Cantab.

WITH NOTES ON THE EYE-SYMPTOMS

By F. Richardson Cross, M.B. Lond., F.R.C.S.

Although instances of abnormally slow pulse are by no means rare, we think that the following case has some special features of interest which justify our recording it; especially as so little is known of the pathology of this phenomenon, that it is desirable to collect as fully as possible the actual facts observed in well-marked cases.

It is generally granted that bradycardia is a symptom of various affections, and not one peculiar to a single disease. These affections may be either extra-cardiac, or those of the heart itself, or there may be a combined lesion such as the arterio-sclerosis of the heart and medulla oblongata postulated by M. Huchard[1]; and there is often extreme difficulty in deciding where the cause of the condition really is, a difficulty which was felt by us in the present case. Most remarkable, too, is the slowness of reaction to cardiac stimulants. Alexander Morison mentions that no effect was noticed for some hours after he injected about twenty minims each of liq. strychninæ and of the tinctures of digitalis and strophanthus, besides nine minims of tincture of belladonna.[2] We noticed a similar lack of response to doses or injections of ether and strychnia, though we could find nothing in the cardiac sounds or the area of dulness to indicate a degenerate muscle, and the rate would rise to the normal of itself after a varying period. Yet in such instances no one can lightly regard the serious alternatives of letting a patient die at once from cerebral asphyxia, or of administering almost fatally strong stimulants. When the heart-beats sink, as in our patient, to 11 or 12 in the minute, it is clear that little is needed to put an end to them altogether. An extremely slow pulse, however, is not always incompatible with prolonged life if due care be taken. Occasionally it seems to be the normal condition in persons who otherwise enjoy good health, and, putting aside the perhaps doubtful cases of Napoleon and Colonel Townshend, instances may be found of individuals who have lived for a long period with pulses of 30 or 40. We know one man now living whose pulse for some time remained about 28.

It is curious how little effect on the general health may be caused by the bradycardia. In the present case there was for a long period neither œdema nor dyspnœa; the colour of the face, the general nutrition, and the mental faculties remained unaltered. He was seen at various times by different consultants, none of whom observed any certain signs of

[1] *Maladies du Cœur*, p 324; quoted by Morison, *Treatment*, 1899, ii. 97.

[2] *Loc. cit.*

cerebral tumour or detected any valvular disease. The notes of the remarkable eye changes, including altitudinal hemianopsia, have been kindly given by Mr. Richardson Cross, whose diagnosis of the brain condition was verified *post mortem.*

The following is a brief history of the case:

A. G., 58, a civil engineer, a man of stout build, looking older than his years, but of temperate habits, had served in the North-Western Provinces of India in early life, and was still engaged in business at home. He was of fairly active habits, and usually enjoyed good health, though we noticed afterwards a gummatous cicatrix in the liver. For ten or twelve years he had complained of failing vision and attacks of giddiness, and showed a marked arcus senilis. In April, 1894, he suffered from sudden vertigo with severe syncopal symptoms, from which he did not recover sufficiently to return to business for some weeks. After this his health was never quite as good as before. Any prolonged strain on the eyes brought on minor attacks of vertigo, but careful and repeated auscultation about this time revealed no valvular lesion or change in the pulse. There was never any trace of albumin in the urine, the lungs were normal, and the liver seemed a little enlarged. In February, 1897, he sent for Mr. Fendick, his usual attendant, early one morning complaining of faintness. His pulse was found to be only 40 beats to the minute; but within two hours it rose to 80, and fluctuated for the rest of the day between 35 and 88. He was kept in bed for some days, and gradually recovered so far as to be able to get out of doors.

On April 19th his pulse was found to be as low as 30 at 8 a.m., and about this time a very slight transient epileptiform convulsion was noticed after each slowing of the pulse. This was at first limited to the muscles of the throat; but later on it spread to the face and upper part of the body, and was accompanied with momentary unconsciousness. Later in the day of the 19th of April the pulse sank to 24, and remained at an average of 30 till 5.30 p.m. on the 20th. It was intermittent, and this increased whenever he attempted to sleep. For some days he remained restless, breathing heavily, with an average pulse of 68. Throughout the illness no difference was observed by either of us between the rate noted at the wrist and over the cardiac region. Scarcely a day passed at this period without epileptiform convulsions more or less severe, sometimes during sleep, and accompanied by a marked slowing of the pulse and intermittency.

On May 13th, at 1.30 p.m., the pulse fell to 24, and by three o'clock rose to 94. During the night there were frequent convulsions, and the pulse fell to 20 for some hours, becoming markedly irregular. The next day the rate fell to 16, and again showed intermittency, rising to the normal on the 15th, but it fell again to 20 the day after. He remained in bed, but fairly well, till the 27th May, when other attacks followed with a rate of 24.

During June he was practically free from trouble; but a fresh series of attacks commenced on the 10th of July, and lasted for some days, with a pulse varying from 20 to 72. The patient then gradually improved, and went to the seaside for some months, returning sufficiently well to resume the management of his business. He persevered with this for the first six months of 1898.

In July the old condition returned; the pulse varied during the latter half of the month from 16 to 80, with a large number of epileptiform attacks of greater or less severity. Many of these occurred immediately upon waking, before he rose in any way from the recumbent posture, and for some days the seizures came on hourly. The mental faculties were perfectly clear, but he was very anxious and apprehensive of approaching death. He took food fairly well, and the body appeared well nourished. The bowels were regular, but he was troubled with considerable flatulency and distension. Occasionally laboured respiration was noticed during sleep. Numerous drugs were tried with little or no success, including various cardiac stimulants, as well as bromides and iodides in large doses, which he had also taken in former years.

He improved for the first ten days of August, the pulse remaining at 70; but he continued weak and very fearful of moving lest another attack should come on. Afterwards mental confusion and wandering began to appear. The pulse fell to an average of 22 on August 11th, and on the 13th it sank to 16. Some difficulty in swallowing was experienced, and definite Cheyne-Stokes respiration followed. After a pause of fifteen seconds, five deep inspirations would be succeeded by twice the number of shallow ones.

On the 15th, after a period of normal pulse-rate, fresh attacks came on, while the heart varied from 15 to 30 for twenty-four hours. Some twitching now occurred in the left hand; the patient began to refuse food, and became excited and slightly delirious. On the 18th the pulse again fell to 22, then to 14, and finally reached 11 beats per minute before midnight. It remained at nearly the same rate for four hours. Respiration was laboured, cold sweats appeared on the face, and neither injections of ether or other measures had any effect. Rectal feeding had to be resorted to, and the urine was drawn off by a catheter on the 19th for the first time. The pulse ranged from 14 to 30. On the 20th several seizures took place; but the pulse improved slightly, and even reached 70 at one time. The patient was evidently sinking; breathing became rapid and the temperature febrile. This was as high as 101.6° on August 21st, when the respiration rose to 60 and the pulse ranged from 25 to 60. He became gradually comatose, and died the same evening.

A necropsy was kindly permitted, and carried out under some difficulties. The body was well nourished; the digestive organs, the kidneys, and the lungs presented nothing of interest except the cicatrix on the liver and some degree of emphysema in the lungs. In the brain there were marked patches of atheroma in the vessels of the base, but otherwise the conditions were normal. No tumour, softening, or trace of hemorrhage could be found. A portion of the medulla was hardened, and examined microscopically. The walls of the vessels were considerably thickened, but no definite changes were observed in the other tissues. The heart was of normal size, and the valves perfect; but the walls showed two or three areas where fibroid degeneration was present. One of these in the ventricular septum near the base formed a nodule readily perceptible to the finger. The aorta was healthy; the coronary arteries were affected by atheromatous degeneration to a slight degree, but there was no thrombus or obliteration of their channels. Microscopically the muscle varied, being quite normal in one place, while in another advanced fibroid changes were present. Thus in part of the septum healthy muscle fibres had almost disappeared.

NOTES BY MR. F. RICHARDSON CROSS.

I saw Mr. A. G. with Mr. Fendick on April 1st, 1886. His right eye was sound and the vision good. In the left there was well-marked optic neuritis, and the very unusual phenomenon of altitudinal hemianopsia. The lower half of the field of vision was lost, central vision was useless. He could see no objects with this eye except by lowering his head and getting them in the upper part of the field, where he could still see them. This was due to a lesion in the upper part of the optic nerve, not to a cerebral condition. Large doses of iodide of potassium somewhat improved the vision. On July 23rd I found the left nerve atrophied. There was loss of the lower half of the field of vision now in both eyes, and the patient saw very badly indeed. The right eye had well-marked optic neuritis.

October 20th.—Both optic nerves showed post-neuritic atrophy; vision—R $\frac{6}{24}$, L $\frac{6}{24}$.

April 19th, 1894. I saw the patient at his house. He had been very ill for a fortnight, with difficulty in walking, giddiness, and nausea. The hemianopsia had scarcely altered. The function of the macula lutea of the right eye was active, that of the left impaired. Each eye saw $\frac{6}{18}$. Double optic atrophy, no neuritis; patient walked with difficulty, the knee-jerks were exaggerated, otherwise all objective symptoms except vision were normal.

April 13th, 1897.—The patient felt very ill. Pupil movements were normal. Vision—R $\frac{6}{24}$, L $\frac{6}{36}$. Hemianopsia as before, optic atrophy, but no fresh neuritis. There was no special sense trouble except the eyesight. The knee-jerks were exaggerated, cardiac weakness with flushing of the face and faintness. I considered that there was no gross cerebral lesion, but defective cerebral circulation and probably degenerate vessels.

REMARKS BY DR. G. PARKER.

I first saw the patient in consultation with Mr. Fendick in July, 1898, and afterwards at his request took charge of the case during his absence. It might be said that in the absence of more definite pathological changes we are left in the dark as to the exact production of the symptoms. The case, however, is clearly one of paroxysmal bradycardia occurring in a patient who had fibroid changes in the ventricles and diseased coronary arteries, while the exciting causes of the attacks were probably changes in the cerebral circulation, eye-strain, and digestive disturbances. Thus, in 1897, I find on three occasions attacks were noted as coming on after reading or using the eyes at his office. In the later stages indeed the tendency to seizure was so strong that it is difficult to discover the exciting causes, but the patient dreaded the slightest noise or excitement for fear of a recurrence. In the classical instance related by Holberton,[1] the heart-rate sank to an average of 33 for some years after an injury of the cord and medulla; but upon an attack of dyspepsia or mental excitement this would fall till it reached even 7½ to the minute, and violent convulsions followed. It was in one of these after-dinner attacks that the patient finally died. Sansom,[2] indeed, seems to think that the influence is often in the other direction, and that the dyspeptic cases seen in paroxysmal and sometimes in persistent bradycardia may be caused by extension to other vagus branches of the disturbance existing in the cardiac area.

Now, bradycardia may be temporary or it may be permanent, or it may be paroxysmal, and the question arises whether any one of these is part of a definite group of symptoms, and always dependent on one and the same cause.

It is clear that permanent bradycardia may be found in perfect health, perhaps more often in very tall men, as Seymour Taylor [3] notices, and again in diseases, such as uræmia and chlorosis, where a high-tension pulse exists. It used to be the fashion to ascribe it to fatty degeneration of the heart, and again to fibroid changes, disease of the coronary arteries, or dilated heart, but all of these may be absent in a well-marked case. Austin Flint regards it as due to a brain lesion, others hold that it is often caused by atheroma of the cerebral vessels. Certainly disease of some part of the nervous system, such as

[1] *Med.-Chir. Tr.*, 1841, xxiv. 76.

[2] *Lancet*, 1897, ii. 523. [3] *Ibid.*, 1891, i. 1247.

an injury to the medulla or cord, is often the only lesion we can discover, and it has been said with some probability that the effect is produced by "an accentuation of inhibitory action coming to the vagus by spinal accessory fibres from the medullary centre" (S. Taylor, *loc. cit.*), since it has been shown that permanent slowing is never caused by the vagus alone.

Temporary bradycardia may also be physiological as in parturition, or it may follow after fevers, in various blood states, or from poisoning. Thus Lewin records a rate of 12 in plumbism. In shock or great pain, and in the last stage of organic heart disease, it is also seen, though how caused is doubtful.

Finally, we may have a paroxysmal form as in this case, with some analogies to paroxysmal tachycardia. Generally, however, there is a permanently infrequent pulse, becoming at times extremely slow, as in Holberton's case. It is very rare to find, as in our patient, a rate of 60, 70, or 80, falling in a short time to 20 or 12, and rising as suddenly. Hodgson's[1] showed an average rate of 29, falling at times to 6, with an occasional gap of thirty seconds, ending in convulsions. This appears to have gone on for six months, after which he recovered and remained fairly well for years. Kinnicutt[2] mentions another with a rate of 30 or 40, falling to 13 and 7; Jacobi one of 12, dropping to 7. R. T. Bruce[3] reported one recently where the pulse was about 20, but for two days before death attacks came on with an interval of forty seconds between the heart-beats followed by convulsions. The breathing showed similar intervals, which curiously resembles Col. Townshend's condition. Adams's celebrated case must be included here.[4] For seven years the rate was usually 30, but a further fall would occur, followed by convulsive seizures, from time to time. There were atheromatous and fatty changes present, but their importance is uncertain.

It is possible that all cases of the Stokes-Adams pheno-

[1] *Brit. M. J.*, 1891, i. 760.
[2] *Tr. Ass. Am. Physicians*, 1889, iv. [3] *Scottish M. & S. J.*, 1898, ii. 330.
[4] *Dublin Hosp. Rep.*, 1827, iv. 353.

menon have this temporary fall of rate, but many reports omit the pulse-rate before and after the seizures. I find only the permanent rate given by Stokes himself,[1] so that it is impossible to say whether his instances were paroxysmal or not.

The phenomena in question may vary from simple vertigo or syncope to strong convulsions and prolonged unconsciousness. True epilepsy is occasionally followed by a very slow pulse; but these convulsions are caused by the slowing through the cerebral anæmia induced by it. One of Stokes's patients could stop them by going on his hands and knees and hanging down his head — the Indian fashion of stopping a fainting attack.

Another point is the long duration of these cases. Mr. Fendick noted pulse-changes for two years and vertigo for ten, and I have given other instances of even longer periods above. Hodgson's patient was alive four years after the attacks, and seems to have recovered from them entirely, though the pulse remained at about 32. The combination of Cheyne-Stokes breathing with paroxysmal bradycardia has been referred to both in our own history and in Bruce's, while Seymour Taylor mentions an instance where the same rhythm was seen in the pulse as in the breathing, and passed away when that improved. Stokes supplies no data as to the pulse in the case which has given the name to the affection, though it appears in his paper on slow pulses. As the combination is so rare, I cannot think that any strong argument can be based on it in favour of the cause of paroxysmal bradycardia lying in the central nervous system, though on the whole the evidence seems to point in that direction. Grasset and others[2] assert that the Stokes-Adams phenomenon may be due to changes in the cerebral vessels alone. Cardiac disease probably plays a part in some cases. Thus the fibroid infiltrations in our case are curiously like those detailed by Ogle[3] in four instances of bradycardia, one of which

[1] *Dublin Q. J. M. Sc.*, 1846, ii. 73.

[2] Clifford Allbutt's *System of Medicine*, 1899, vol. vi., p. 341.

[3] *Lancet*, 1897, i. 296.

suffered from convulsions. Masses of fibroid infiltration were found in the septum and bases of the ventricles; but there is, after all, no proof that the intra-cardiac ganglia were weakened by them, and that unusual effect was thus given to the inhibitory impulses of the vagus. Such changes are, I think, found in many cases where the pulse is unaltered.

### A CASE OF DISLOCATION OF THE HAND AND CARPUS BACKWARDS.

